# Primary Fallopian Tube Cancer in an 89-Year-Old Patient

**DOI:** 10.1155/2021/2870057

**Published:** 2021-10-08

**Authors:** Emmanuel N. Kontomanolis, Antonios Koutras, Thomas Ntounis, Michail Diakosavvas, Kyveli Angelou, Athina A. Samara, Themistoklis Grigoriadis, Pelagia Kadari, Ioannis Tsirkas, Marianna Theodora, Zacharias Fasoulakis

**Affiliations:** ^1^Democritus University of Thrace, Alexandroupoli, Greece; ^2^National and Kapodistrian University of Athens, Athens, Greece; ^3^University Hospital of Larissa, Greece

## Abstract

Fallopian tube cancer is an extremely rare gynecological condition, accounting for just 1 to 2% of all female tract malignancies. The mean age of diagnosis is similar to that of ovarian cancer, between 60 and 75 years, but it can affect a wide spectrum of ages. Advanced age and family history of ovarian and breast cancer are the main risk factors, since they are associated with increased incidence of this uncommon entity. In this study, we report a rare case of an elderly, 89-year-old patient that presented to our clinic due to vaginal bleeding.

## 1. Case Report

**Figure 1:**
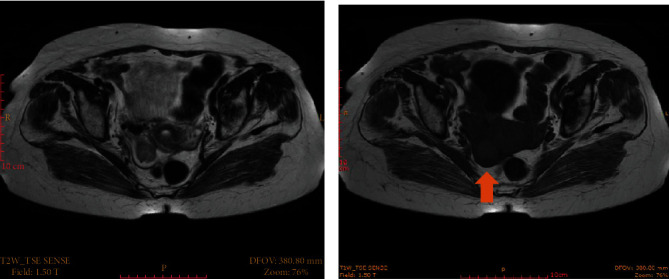
(a) Transaxial T2-weighted MRI shows a right adnexal mass (arrow) displaying thick wall with high signal intensity. (b) Loss of signal on T1-weighted image.

An 89-year-old female subject was referred to our Outpatient Clinic with traces of vaginal bleeding and a smelly yellowish discharge. No medication was reported in reference to bleeding disorders. Her level of communication was perfect while no dementia was observed during clinical examination. She reported vaginal delivery twice, while previous medical history reported no major surgeries except for a cholecystectomy. No comorbidities or allergies were reported. She was on medication for hyperthyroidism. The biomarker carbohydrate antigen 125 (CA-125) is a glycoprotein which is normally detected in the sera of patients suspicious of uterine tube cancer. In our patient, it was increased in two consecutive measurements. The rest of the tumor markers (*α*-FP, Ca 15.3, Ca 19.9, and CEA) were within normal range. Vaginal ultrasound and MRI imaging showed a tube-like structure in the vicinity of the right ovary (Figure 1).

An exploratory laparotomy revealed the solid tumor of the right tube, measuring 28/30 mm. Both ovaries, the bladder, and the contralateral horn were free of any gross lesions. 300 mL of ascites fluid was aspirated. A tumor biopsy of 28/30/20 mm was collected and sent to frozen section for histology. A total hysterectomy with bilateral pelvic and para-aortic lymph node dissection was performed. Parietal and peritoneal biopsies were done. The postoperative course was uneventful. The patient was discharged on day 12 post-op.

**Figure 2:**
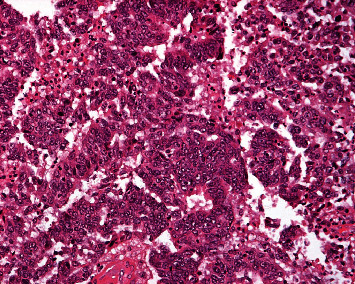
Cystic and solid mass with micropapillary configuration.

Macroscopically, the tube was enlarged and occupied by partially solid and partially cystic tumor, which was filling the lumen. The maximum diameter of the tumor was 6 cm. The cut surface had white gray appearance with solid and micropapillary configuration (Figure 2). Hemorrhage and necrosis were also noted. Ovaries, uterus, and fimbriae were free. Fallopian tube surface was massively occupied by the neoplasm.

Histologically complex branches of micropapillae, solid masses of cuboidal cells with eosinophilic cytoplasm, and slit-like spaces (fusion of papillae) were the main patterns of growth. Moreover, glandular and cribriform patterns were common. The cells had nuclear pleomorphism with prominent nucleoli, high mitotic index, and apoptotic bodies. Serious tubal intraepithelial carcinoma (STIC) adjacent to infiltrative carcinoma was seen. Lymphovascular invasion with surface involvement was observed. The patient was diagnosed with primary tubal high-grade serous cancer, staged pT1cN2.

## 2. Discussion

Primary fallopian tube carcinoma (PFTC) is the least prevalent cancer of the female reproductive system. The first microscopic description of this malignancy was in 1861 by Rokitansky. In 1888, Orthman published the first report on the condition [1]. PFTC was first described in 1897 by Renaud [2]. PFTC has close similarity with other types of cancer. Firstly, it is extremely challenging to distinguish between PFTC and primary peritoneal serous carcinoma or serious epithelial ovarian cancer during and after surgery. Secondly, it has a clinical and histological resemblance with epithelial ovarian cancer (EOC) [3].

PFTC causes tubal distension, which then presents as abdominal pain. This characteristic allows for the diagnosis of PFTC at early stages compared to EOC, which is often diagnosed at advanced stages [4]. The etiology of PFTC remains unclear. The carcinoma is common in women between 17 and 79 years (mean age of 55 years) [5]. Furthermore, it has a peak incidence at the age of between 60 and 64. Most patients who develop PFTC are postmenopausal. However, the risk for PFTC is lower in pregnant mothers, women with high parity, and those who use oral contraceptives [6].

PFTC does have specific clinical signs or symptoms. However, most of the patients present with abdominal pain, which may be dull or colicky due to peristalsis within the fallopian tube or its distension. In other cases, patients experience vaginal watery discharge or bleeding [7]. The condition has three classic symptoms according to Latzko's triad. They include pelvic or abdominal growth; colicky abdominal pain, which improves when the patient experiences vaginal discharge; and intermittent profuse serosanguinous vaginal bleeding. This triad is applicable in 15% of patients with PFTC cases [8]. The case selected for this paper was a postmenopausal woman without any significant predisposing factor. She did not have pelvic inflammatory disease, nulliparity, or subfertility. However, the patient had colicky abdominal pain (which subsided after a vaginal discharge), an abdominal growth, and serosanguinous bleeding from the vagina that was profuse.

Hu et al. were the first people to establish the criteria for diagnosing PFTC. Todays criteria represents a modification of Hu's criteria by Seldis et al. criteria slightly to what it is now. The criteria outline the features a case must meet before qualifying as PFTC. Firstly, the main tumor must originate from the endosalpinx. Secondly, the tumor must have a histological pattern, which must reproduce the epithelium of the tubal mucosa. Thirdly, there should be evidence demonstrating the evolution of the tubal epithelium from benign to malignant. Lastly, the patient's endometrium and ovaries should be normal. If not so, they should have tumors that are smaller than the one within the tube [9, 10].

It is very rare for clinicians to diagnose PFTC in the preoperative period [11]. Just 0–10% of available PFTC cases underwent diagnosis preoperatively. Clinicians miss a further 50% of PFTC cases in the intraoperative period [7]. Clinicians can address this challenge by considering PFTC as a differential diagnosis for patients experiencing unusual per vaginal bleeding, vaginal discharge, or spotting, which has a negative diagnostic curettage. Pap smear can also be crucial in confirming PFTC since it can show positivity in 10-36% of PFTC cases [8]. Ca-125 is equally crucial in the prognosis of PFTC. It is a tumor marker, which helps in diagnosing and monitoring response to PFTC treatment. Approximately 80% of PFTC patients exhibit increased levels of pretreatment Ca-125 in their blood [11]. Ca-125 also helps in detecting tumor recurrence during follow-up.

Imaging studies can also help diagnose suspected gynecological malignancies. An endovaginal and a transabdominal ultrasound is the easiest of them all. They allow clinicians to examine the abdomen and the genital tract for any growths as part of the initial investigation. Other available radiological studies are computed tomography (CT) scan and magnetic resonance imaging (MRI). A cancerous lesion may appear as a lobular, small, and solid mass on a scan (MRI or CT). Clinicians can predict PFTC when they visualize a solid papillary intratubal mass on a CT scan. However, ultrasound does not give specific results, not tubal carcinoma. Instead, the results resemble those of other pelvic illnesses like tube-ovarian abscess and ovarian tumor. In such cases, clinicians can use other diagnostic approaches to determine the existence of cancer. For instance, an echogram can help diagnose PFTC by showing a mass that is sausage-like in shape. The mass may also be cystic but with mural nodules and spaces, or multilobular but with a cog-and-wheel appearance [12, 13]. It is, however, important to note that MRI is better in detecting any infiltration of extramural organs with tumors compared to CT scan or ultrasound [14].

Malignant epithelial tumors account for almost 90% of all ovarian cancers. The carcinomas fall into at least five types based on immunohistochemistry, molecular genetic analyses, and histopathology. They include endometrioid carcinoma (EC), which accounts for 10% of carcinomas, and mucinous carcinoma (MC-3%). The others are high-grade serous carcinoma (HGSC-70%), clear-cell carcinoma (CCC-10%), and low-grade serous carcinoma (LGSC: <5%) [15].

The best approach for treating PFTC is surgery. PFTC surgical treatment focuses on removing the tumor as much as possible similar to what cytoreductive surgery of ovarian carcinoma does. Surgeons use various approaches to accomplish this goal, depending on the extent of the carcinoma. The most common procedures are hysterectomy (total or selective) with bilateral salpingo-ovariectomy, lymphadenectomy (pelvic and para-aortic) for fallopian tube carcinomas of any stage, and omentectomy [16]. Patients then undergo adjuvant chemotherapy (which is platinum based) in the postoperative period (like EOC patients) to eliminate any remaining cancerous cells. Postoperative radiotherapy is also used at times, but its role is less clear [17].

This treatment approach has shown a response rate of 53-92%. However, the prognosis of PFTC may differ depending on various factors. The first and most important factor is the stage at which the carcinoma is diagnosed [6]. Others are ascites, the remaining size of the tumor after cytoreduction, and the histologic grade of the tumor [3].

In conclusion, PFTC is an uncommon form of cancer of the female reproductive system. It comprises <1% of all malignancies of the female reproductive system. It has close histological and clinical similarities with ovarian epithelial carcinoma. The diagnosis of PFTC in the preoperative period is challenging because the course of the neoplasm is usually silent. Instead, a pathologist first appreciates it at the time of operation. Most cases of PTFC do not exhibit the pathognomonic symptom complex of hydrops tubal profluence. For this reason, clinicians diagnose PFTC by conducting a differential diagnosis on women in the peri- and postmenopausal age presenting with unusual bleeding of the uterus, abnormal cervical smear, adnexal growth, pelvic pain, and complicated PID. PTFC has a treatment approach which resembles that of ovarian cancer. It entails omentectomy, dissection of bilateral pelvic and para-aortic lymph nodes, and total abdominal hysterectomy with bilateral salpingo-oophorectomy [18].
